# Stromal Cell Derived Factor‐1 Promotes Hepatic Insulin Resistance via Inhibiting Hepatocyte Lipophagy

**DOI:** 10.1111/jcmm.70352

**Published:** 2025-01-24

**Authors:** Chunfeng Lu, Yuting Zhang, Cuilian Sun, Yuhang Na, Haotian Sun, Jianhua Ma, Xueqin Wang, Xiaomin Cang

**Affiliations:** ^1^ Department of Endocrinology Secondary Affiliated Hospital of Nantong University and the First People's Hospital of Nantong Nantong Jiangsu China; ^2^ Department of Pathology Lixiang Eye Hospital of Soochow University Suzhou Jiangsu China; ^3^ Department of Pathogen Biology, Medical College Nantong University Nantong Jiangsu China; ^4^ Department of Endocrinology, Nanjing First Hospital Nanjing Medical University Nanjing Jiangsu China

**Keywords:** hepatic insulin resistance, hepatocyte, lipophagy, stromal cell derived factor‐1

## Abstract

Saturated fatty acid (SFA) accumulation in liver decreases hepatocyte lipophagy, a type of selective autophagy that degrades intracellular lipid droplets, leading to hepatic insulin resistance (IR), which contributes to simultaneous increases in liver glucose production and fat synthesis, resulting in hyperglycemia and dyslipidemia traits of type 2 diabetes mellitus (T2DM). Stromal cell derived factor‐1 (SDF‐1), a cytokine produced by hepatocytes, inhibits autophagy. In this study, we evaluated the hypothesis that SDF‐1 promoted hepatic IR via inhibiting hepatocyte lipophagy during T2DM. Furthermore, we probed the downstream pathway participating in the role of SDF‐1. The results showed that the neutralising of SDF‐1 improved hepatic IR via promoting hepatocyte lipophagy in a mouse high‐fat and high sucrose diet (HFHSD)‐induced T2DM model. In vitro, SDF‐1 expression and release increased in palmitic acid (PA, a kind of SFA)‐treated hepatocytes. Meanwhile, SDF‐1 bound to up‐regulated C‐X‐C chemokine receptor type 4 (CXCR4) and C‐X‐C chemokine receptor type 7 (CXCR7) on PA‐treated hepatocytes. Subsequently, SDF‐1 inhibited lipophagy in PA‐treated hepatocytes via CXCR4, rather than CXCR7. Finally, SDF‐1/CXCR4/protein kinase B (AKT)/mechanistic target of rapamycin (mTOR) pathway‐inhibited lipophagy promotes PA‐induced hepatocyte IR. Collectively, this study discovered that SDF‐1 might inhibit lipophagy in SFA‐treated hepatocytes to promote hepatic IR via CXCR4/AKT/mTOR pathway.

## 1 Introduction

Fatty acids are major components of biological cell membranes and play important physiological roles in energy storage, membrane structure, intracellular signalling and transcriptional regulation of genes. However, chronic exposure to fatty acids can lead to severe pathophysiological conditions such as type 2 diabetes mellitus (T2DM) by depositing excess fat in non‐adipose tissues such as liver [1]. It has been shown that diets rich in saturated free fatty acids (FFAs) induce a greater increase in liver fat and insulin resistance (IR) compared to unsaturated FFAs [2]. Liver is the primary organ regulating glucose and lipid homeostasis and long‐term exposure to saturated FFAs such as palmitic acid (PA) has been shown to induce hepatic IR [3]. Hepatic IR contributes to simultaneous increases in liver glucose production and fat synthesis, resulting in hyperglycemia and hyperlipidemia features of T2DM [4].

Though the mechanisms of hepatic IR are not entirely clear, disturbances in lipophagy contribute to hepatic IR. Lipophagy, the latest subtype of autophagy to be identified, is the selective degradation process of lipid droplets by autophagy [5]. Hepatic ceramide reduction ameliorates hepatic IR via promoting lipophagy in mouse alcoholic steatosis model [6]. Our previous study also reveals that chromosome 9 open reading frame 72 (C9orf72)‐inhibited lipophagy facilitates hepatic IR in a mouse T2DM model [7].

Stromal cell derived factor‐1 (SDF‐1), also named C‐X‐C motif chemokine ligand 12 (CXCL12), is a cytokine produced by hepatocytes [8]. SDF‐1 binds to its two receptors C‐X‐C chemokine receptor type 4 (CXCR4) [9] and C‐X‐C chemokine receptor type 7 (CXCR7) [10], G‐protein coupled receptors on the surface of hepatocytes [11]. SDF‐1 plays an inhibitory role on autophagy. Inhibition of SDF‐1 and its receptor CXCR4 increases utero‐placental autophagy [12]. SDF‐1/CXCR4 negatively regulates autophagy in human neuroblastoma H4 cells under normal nutritional conditions [13]. This study aimed to test the hypothesis that SDF‐1 exacerbated hepatic IR via inhibiting lipophagy in hapatocytes. First, the therapeutic role of the modulation of SDF‐1 on hepatic lipophagy and IR in a T2DM mouse model was identified. Then the production of SDF‐1 in PA‐treated mouse primary hepatocytes was detected. Next, whether SDF‐1 bound to CXCR4 and CXCR7 on hepatocytes in an autocrine manner was determined. Additionally, the function and mechanism of SDF‐1 production on the lipophagy of PA‐challenged hepatocytes were analysed. Finally, the effect of SDF‐1‐regulated lipophagy on the IR of PA‐stimulated hepatocytes was detected.

## 2 Materials and Methods

### 2.1 Animal Experiments

Animal experiments were carried out in accordance with the guidelines of China for the care and use of animals and were permitted by the ethics committee of Nantong University (No. 220240180). Male C57BL/6J mice (3 weeks old) were obtained from the Laboratory Animal Center of Nantong University and allowed to acclimatise to the clean grade environment for 1 week before the experiments. Four‐week‐old mice were then fed a standard diet (SD; D10010501, Research Diets, USA) or a high‐fat and high sucrose diet (HFHSD; D11725; Research Diets) for 16 weeks to induce T2DM model. The mice were assigned into normal, T2DM, T2DM + SDF1 neutralising antibody (MAB310, R&D Systems, USA; 1 mg/kg/day, intrahepatic injection), T2DM + SDF1 neutralising antibody +3‐Methyladenine (3‐MA; autophagy inhibitor; HY‐19312, MedChemExpress, USA; 30 mg/kg/day, intrahepatic injection), T2DM + metformin (MET; S5958, Selleck, USA; 250 mg/kg/day, once a day from week 4 to week 6 of HFHSD feeding, oral administration) groups. The intrahepatic injection was performed once a day from week 15 to week 16 of HFHSD feeding. MET was used as a positive T2DM treatment control.

### 2.2 Immunofluorescence

Immunofluorescence (IF) was performed on frozen sections. Tissues were embedded in optimal cutting temperature compound (OCT; 4583, Sakura Finetek, Japan) after fixing, and 7‐μm‐thick sections were mounted on the slides using the frozen section machine (CM1860, Leica Biosystems, USA). The sections were boiled in 10 mM citric acid (C9999, Merck, USA) at pH 6.0 for 5 min, and then were exposed to 1% bovine serum albumin (BSA; 10711454001, Merck) in phosphate‐buffered saline (PBS) containing 0.1% Tween‐20 (P9416, Merck) to block non‐specific sites. Then the sections were incubated with primary antibodies against albumin (rabbit, ab207327, Abcam; 1:100), SDF‐1 (mouse, sc‐74271, Santa Cruz Biotechnology, USA; 1:50) and LC3 (mouse, 83506, Cell Signalling Technology, USA) overnight at 4°C. Then, the sections were rinsed with PBS 3 times for 5 min each and stained with secondary antibodies Alexa Fluor 594 goat anti‐rabbit IgG (ab150080, Abcam; 1:200) and Alexa Fluor 488 goat anti‐mouse IgG (ab150113, Abcam; 1:200) for 1 h at room temperature (RT). The slide was then counterstained with 4′,6‐diamidino‐2‐phenylindole (DAPI; D9542, Merck).

The co‐localization of autolysosome and lipid droplet (LD) in hepatocytes were identified. The hepatocytes were co‐labelled with 50 nM Lyso‐Tracker Red (L7528, Thermo Fisher Scientific, USA; autolysosome staining) and 5 μg/mL BODIPY 493/503 (D3922, Thermo Fisher Scientific; LD staining) followed by incubation in 0.2 μg/mL DAPI. Then, the hepatocytes were embedded in Mowiol 4–88 (81381, Merck). The Pearson correlation coefficient (PCC) was used to quantitatively analyse the co‐localization of albumin/SDF‐1 and autolysosome/LD [14]. Images were acquired using an Olympus IX70 fluorescence microscope (Japan) and quantified using ImageJ software.

### 2.3 Fasting Blood Insulin and Glucose Measurements

Fasting blood insulin (ab277390, Abcam) and glucose (ab65333, Abcam) levels from mice (at the age of 20 weeks) fasting for 12 h, were determined using enzyme‐linked immunosorbent assay (ELISA) kits. The homeostasis model of assessment‐insulin resistance (HOMA‐IR) was calculated as follow: HOMA‐IR = [fasting blood glucose levels (mmol/L)] × [fasting serum insulin levels (μIU/mL)]/22.5.

### 2.4 Mouse Liver Index

The mice were sacrificed by cervical dislocation immediately following blood sample collection. The mouse liver tissues were collected and weighed. The liver index was calculated as follow: Liver index (mg/g) = liver weight (mg)/animal weight (g).

### 2.5 Haematoxylin and Eosin Staining

The mouse liver tissues were fixed in 4% paraformaldehyde (PFA) at 4°C overnight, performing on 4‐μm‐thick sections using a Haematoxylin and Eosin (HE) stains kit (ab245880, Abcam), stained with haematoxylin for 3 min and eosin for 20 s at RT. Images were captured using the PerkinElmer Automated Quantitative Pathology System.

### 2.6 Oil Red O Staining

For frozen sections, liver tissues were fixed with 4% PFA overnight and then infiltrated in 30% sucrose. The tissues were embedded in OCT compound and stored at −80°C until analysis. Frozen tissues were sectioned at 6 μm using a cryostat. The sections were air‐dried for 30 min and fixed in 10% formalin for 5 min. The slides were washed with 60% isopropanol and then stained with 60% isopropanol solution containing 0.5% oil red O (O1391, Merck) for 15 min. Then the slides were counterstained with haematoxylin and washed with distilled water.

Oil red O staining was performed to detect lipid accumulation in hepatocytes. In brief, the treated hepatocytes were washed with PBS, fixed with 4% PFA for 5 min, and then incubated in 60% isopropanol solution containing 0.5% oil red O for 30 min. Finally, the hepatocytes were observed and photographed under a microscope (BX63, Olympus).

### 2.7 Primary Mouse Hepatocyte Isolation, Culture, and Treatment

The isolation and culture of primary mouse hepatocytes were performed as previous description [15]. The primary mouse hepatocytes were treated with PA (S3794, Selleck, USA; 0.5 mmol/L for 24 h), SDF‐1 neutralising antibody (1 μg for 24 h), AMD3100 (CXCR4 antagonist; HY‐10046, MedChemExpress, USA; 10 μM for 24 h), ACT‐1004‐1239 (CXCR7 antagonist; HY‐142617, MedChemExpress; 6 nM for 24 h), MK‐2206 2HCl (AKT inhibitor; HY‐10358, MedChemExpress, 10 μM for 24 h), XL388 (mTOR inhibitor; HY‐13806, MedChemExpress, 100 nM for 24 h), SC79 (AKT activator; HY‐18749, MedChemExpress, 4 μg/mL for 24 h), MHY1485 (mTOR activator; HY‐B0795, MedChemExpress, 5 μM for 24 h), or 3‐MA (10 mM for 24 h).

### 2.8 Western Blot

Total protein was extracted from hepatocytes using radioimmunoprecipitation assay (RIPA) lysis buffer (89901, Thermo Fisher Scientific) supplemented with a protease and phosphatase inhibitor cocktail (78438, Thermo Fisher Scientific) and quantified using a BCA protein assay kit (A55860, Thermo Fisher Scientific). Proteins were separated by sodium dodecyl sulfate‐polyacrylamide gel electrophoresis (SDS‐PAGE) and transferred onto polyvinylidene fluoride (PVDF) membranes. The membranes were blocked with 5% BSA for 1 h at RT and were then incubated with primary antibodies, including mouse SDF‐1, mouse CXCR4 (sc‐53534, Santa Cruz Biotechnology), rabbit CXCR7 (ab117836, Abcam), mouse LC3, rabbit p62 (ab91526, Abcam), rabbit ATG7 (2631, Cell Signaling Technology), mouse p‐AKT (S473, 66444‐1‐Ig, Proteintech, USA), mouse AKT (AHO1112, Thermo Fisher Scientific), rabbit p‐mTOR (S2448, 44‐1125G, Thermo Fisher Scientific), rabbit mTOR (PA5‐34663, Thermo Fisher Scientific), mouse GAPDH (MA5‐15738, Thermo Fisher Scientific), rabbit Na/K ATPase (3010, Cell Signaling Technology) at 4°C overnight. The membranes were washed three times with tris‐buffered saline with 0.1% Tween‐20 (TBST) and were then incubated with the corresponding secondary antibodies, including horseradish peroxidase (HRP)‐conjugated goat‐anti‐mouse IgG (31430, Thermo Fisher Scientific) and HRP‐conjugated goat‐anti‐rabbit IgG (31460, Thermo Fisher Scientific), at RT for 2 h. Each antibody was used as the concentration of 1 μg/mL unless otherwise specified. Protein bands were visualised with a chemiluminescent HRP substrate kit (15159, Thermo Fisher Scientific). Band intensity was measured using ImageJ software.

### 2.9 Elisa

The SDF‐1 protein levels in hepatocyte culture supernatant were detected using a mouse SDF‐1 ELISA kit (KE10049, Proteintech) according to the manufacturer's instructions.

### 2.10 The Extraction of Hepatocyte Plasma Membrane Fraction

The plasma membrane fractions from hepatocytes were extracted using the plasma membrane protein extraction kit (ab65400, Abcam) according to the manufacturer's instructions. The plasma fraction was subjected to SDS‐PAGE and analysed by Western blot. Na/K ATPase was used as the marker of the plasma membrane fraction.

### 2.11 Immunoprecipitation

The interaction of endogenous SDF‐1/CXCR4 and SDF‐1/CXCR7 was detected by Immunoprecipitation (IP). The hepatocytes were lysed with IP buffer (87787, Thermo Fisher Scientific) supplemented with protease and phosphatase inhibitors cocktail. The lysate was incubated overnight at 4°C with rabbit SDF‐1 (ab25117, Abcam), mouse CXCR4, or rabbit CXCR7 (PA5‐27077, Thermo Fisher Scientific) antibody. On the following day, the lysate was incubated with protein G beads (10003D, Thermo Fisher Scientific), followed by slow rotation at 4°C for 8 h, and then Western blot.

### 2.12 Detection of Glucose Levels in Hepatocyte Culture Medium and Analysis of Glucose Consumption

Two days before the experiments, the hepatocytes were plated into 96‐well culture plates with some wells left blank. After the cells reached confluence, the medium was replaced by DMEM supplemented with 0.2% BSA. After 12 h, the medium was removed and the same BSA Dulbecco's Modified Eagle Medium (DMEM) containing PA or other reagent was added to all wells including the blank wells. After 24 h, the medium was removed, and its glucose concentrations were determined using a glucose oxidase kit (MAK097, Merck). The amount of glucose consumption (GC) was calculated by the glucose concentrations of blank wells subtracting the remaining glucose in cell plated wells.

### 2.13 Statistical Analyses

The data were presented as mean ± SEM. Shapiro–Wilk's test and Levene's test were used to examine assumptions of normality and homogeneity of variance, respectively. Statistical analysis was performed using GraphPad Prism 8 software. Significance was assessed by one‐way analysis of variance (ANOVA) followed by Dunnett's test for the comparisons of multiple means within an experiment. *p* value less than 0.05 was considered statistically significant.

## 3 Results

### 3.1 The Neutralising of SDF‐1 Mitigates Hepatic IR via Promoting Hepatocyte Lipophagy in T2DM Mice

**FIGURE 1:**
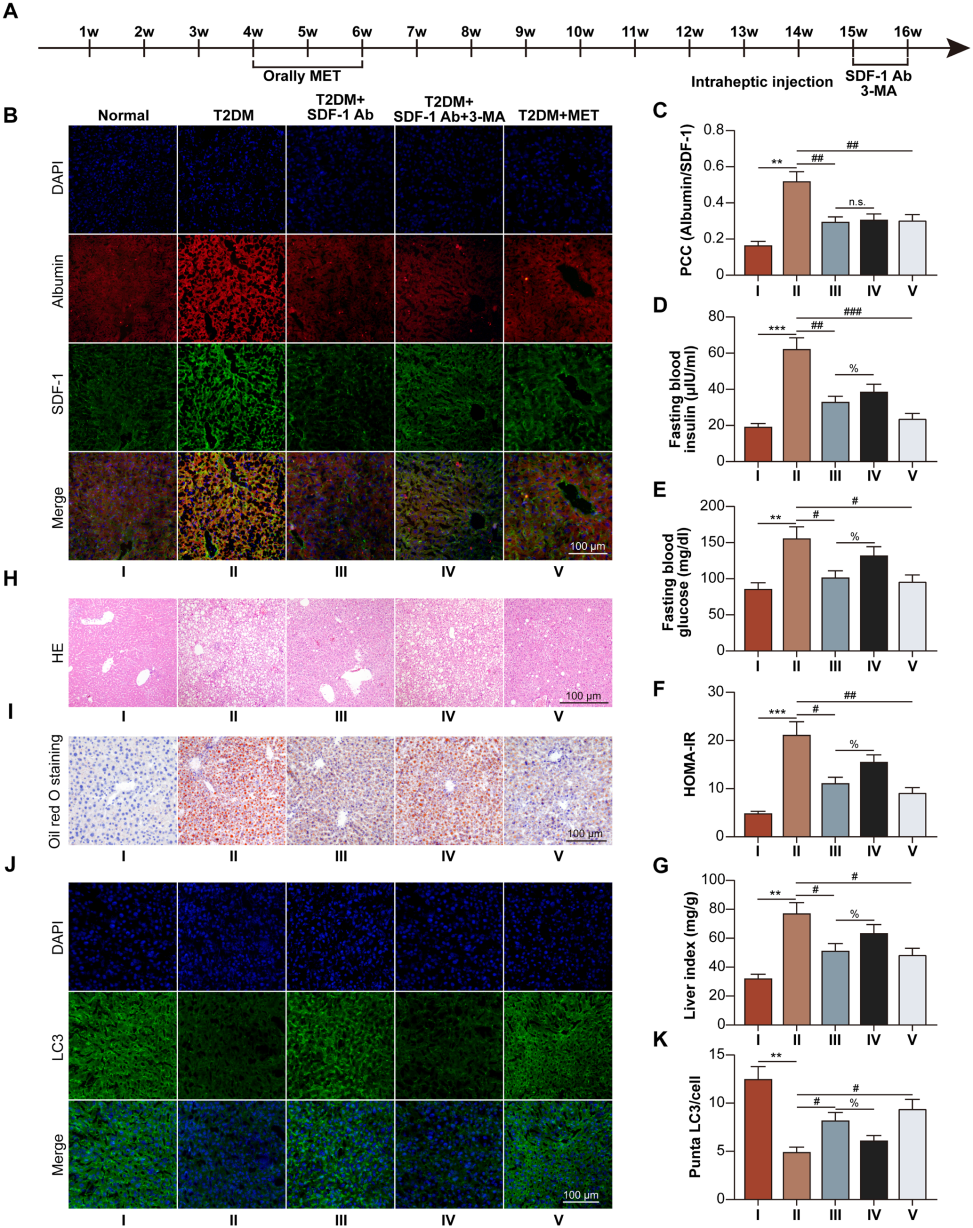
The neutralising of SDF‐1 mitigates hepatic IR via promoting hepatocyte lipophagy in T2DM mice. The mice were assigned into normal, T2DM, T2DM + SDF‐1 neutralising antibody (1 mg/kg, intrahepatic injection), T2DM + SDF‐1 neutralising antibody +3‐MA (30 mg/kg, intrahepatic injection), and T2DM + MET (250 mg/kg/day, oral administration) groups. (A) The experimental flow was shown. (B) Hepatocyte marker albumin (green) and SDF‐1 (red) were labelled in mouse liver tissues. (C) The co‐localization of albumin and SDF‐1 was analysed. (D) Fasting blood insulin levels were detected. (E) Fasting blood glucose levels were detected. (F) HOMA‐IR was calculated. (G) The liver index was analysed. (H) Representative images of mouse livers were shown. (I) Liver oil red O staining was shown. (J) The autophagy marker LC3 (green) was labelled in mouse liver tissues. (K) The puncta LC3 per cell was analysed. **p < 0.01, ***p < 0.001 versus normal group. #*p* < 0.05，##*p* < 0.01，###*p* < 0.001 versus T2DM group. **p < 0.01 versus T2DM + SDF‐1 neutralising antibody group. The n.s. stood for no significance.

The timeline of the animal experiment was illustrated in Figure 1A. We constructed a mouse T2DM model by HFHSD administration for 16 weeks. The co‐localization of hepatocyte marker albumin and SDF‐1 increased in T2DM mice. SDF‐1 neutralising antibody or MET reduced the co‐localization, while autophagy inhibition by 3‐MA unaffected the co‐localization (Figure 1B,C). Consequently, fasting blood insulin, fasting blood glucose, and HOMA‐IR increased in the T2DM group compared with those of the normal group. SDF‐1 neutralising antibody or MET led to deceased insulin, glucose, and HOMA‐IR, while 3‐MA reversed down‐regulation caused by SDF‐1 neutralisation (Figure 1D–F). Moreover, the liver index and the accumulation of LD increased in the T2MD mice. SDF‐1 neutralisation or MET treatment decreased the liver index and the accumulation of LD, while 3‐MA reversed the effects of SDF‐1 neutralisation (Figure 1G–I). The intensity of LC3 puncta fluorescence in hepatocytes (Figure 1J,K) was high in normal group, decreasing in T2DM group, while SDF‐1 neutralising antibody increased LC3 puncta fluorescence, which was inhibited by 3‐MA. MET also increased LC3 puncta fluorescence in T2DM hepatocytes.

### 3.2 SDF‐1 Expression and Release Increase in PA‐Treated Hepatocytes

**FIGURE 2:**
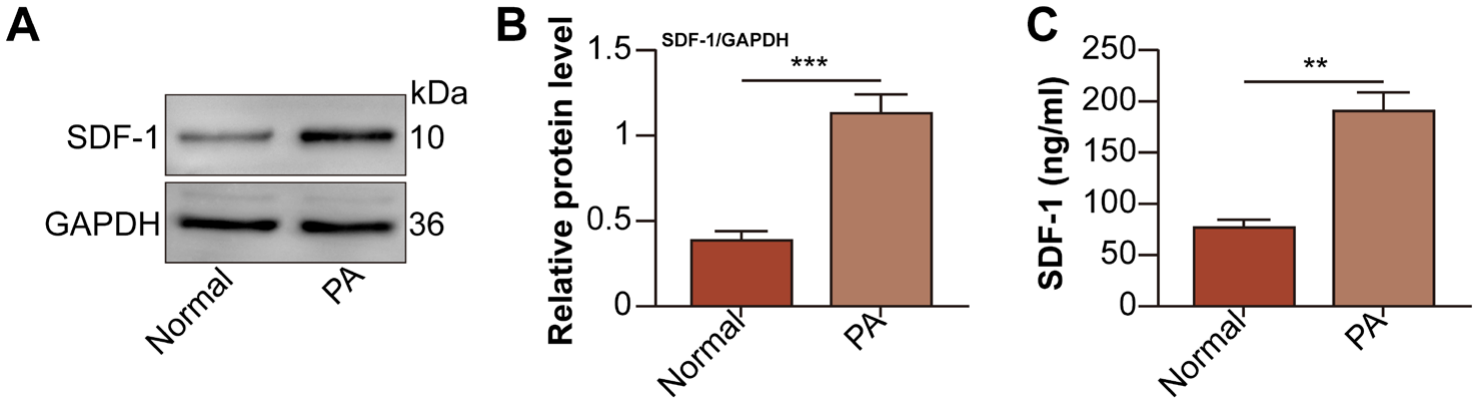
SDF‐1 expression and release increase in PA‐treated hepatocytes. The hepatocytes were divided into normal and PA (0.5 mmol/L for 24 h) groups. (A) SDF1 protein levels in hepatocytes were detected by Western blot. (B) SDF‐1 relative protein levels were analysed. (C) SDF‐1 protein levels in hepatocyte culture supernatant were measured by ELISA. **p < 0.01, ***p < 0.001 versus normal group.

SDF‐1 protein levels in normal and PA‐treated hepatocytes were evaluated by Western blot. SDF‐1 expression was higher in PA‐treated hepatocytes than that of normal hepatocytes (Figure 2A,B). Meanwhile, the release of SDF‐1 from PA‐treated hepatocytes to the culture medium also increased (Figure 2C). These results implied that the up‐regulation of SDF‐1 expression and release might be associated with PA‐induced hepatocyte IR.

### 3.3 CXCR4 and CXCR7 Expression Increase on PA‐Treated Hepatocytes

**FIGURE 3:**
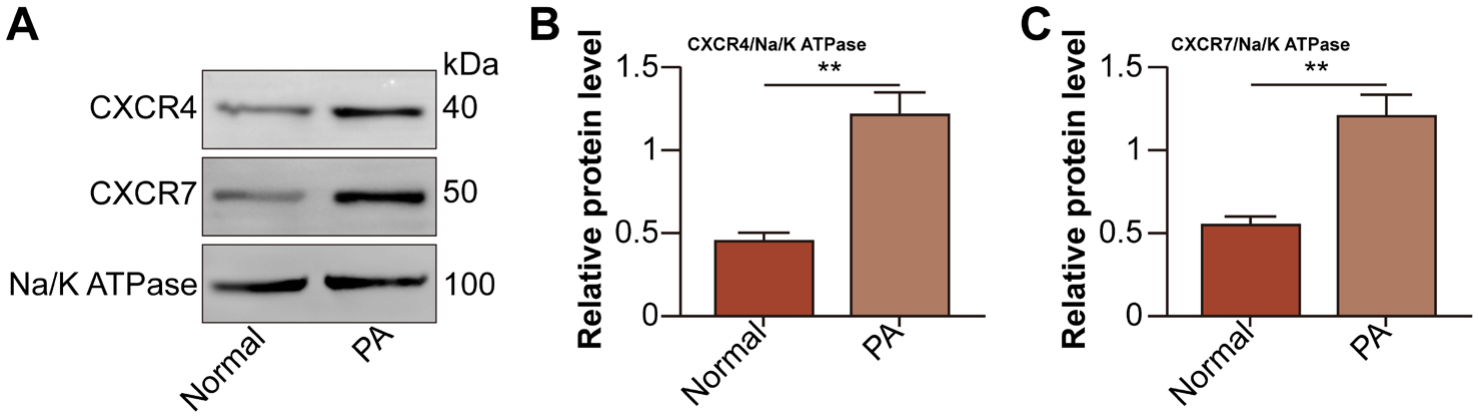
CXCR4 and CXCR7 expression increase on PA‐treated hepatocytes. The hepatocytes were divided into normal and PA (0.5 mmol/L for 24 h) groups. (A) CXCR4 and CXCR7 protein levels on the plasma membrane of hepatocytes were detected by Western blot. (B, C) CXCR4 and CXCR7 relative protein levels were analysed. **p < 0.01 versus normal group.

SDF‐1 binds to its two receptors CXCR4 and CXCR7 on cell plasma membrane to exert its roles [16]. Therefore, CXCR4 and CXCR7 expression on the plasma membrane of hepatocytes were detected. Compared to that of normal group, CXCR4 and CXCR7 expression on PA‐treated hepatocytes was higher (Figure 3A–C).

### 3.4 SDF‐1 Binds to CXCR4 and CXCR7 on PA‐Treated Hepatocytes

**FIGURE 4:**
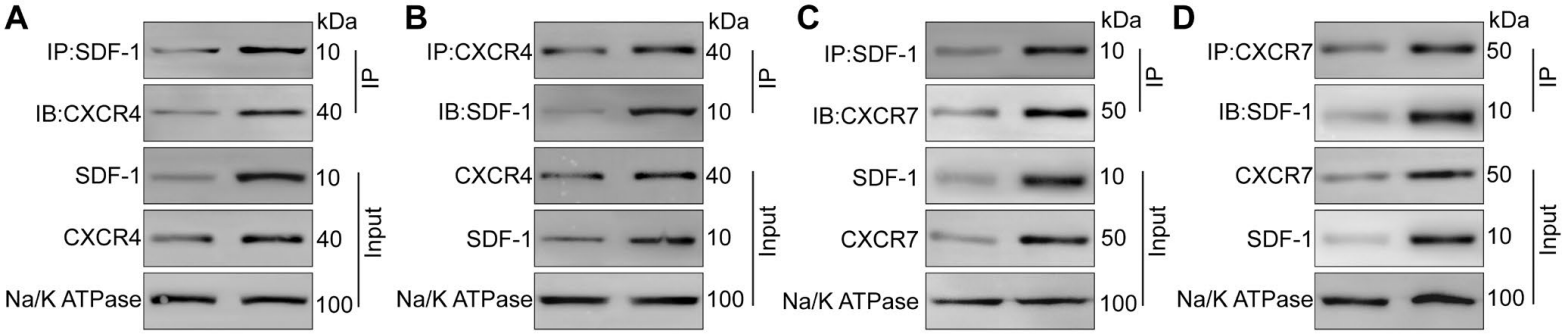
SDF‐1 binds to CXCR4 and CXCR7 on PA‐treated hepatocytes. The hepatocytes were divided into normal and PA (0.5 mmol/L for 24 h) groups. IP was performed to detect the interaction between SDF‐1 and CXCR4 on the plasma membrane of hepatocytes using SDF‐1 (A) or CXCR4 (B) antibody. IP was performed to detect the interaction between SDF‐1 and CXCR7 on the plasma membrane of hepatocytes using SDF‐1 (C) or CXCR7 (D) antibody.

The interaction between SD‐1 and CXCR4 on the plasma membrane of hepatocytes was independent of PA treatment. Additionally, the interaction between SDF‐1 and CXCR4 on the plasma membrane of PA‐treated hepatocytes was stronger than that of normal group (Figure 4A,B). The existence and tendency of the interaction between SDF‐1 and CXCR7 on the plasma membrane of hepatocytes was similar to the interaction between SDF‐1 and CXCR4 (Figure 4C,D).

### 3.5 SDF‐1 Promotes Lipophagy in PA‐Treated Hepatocytes via CXCR4, Rather Than CXCR7

**FIGURE 5:**
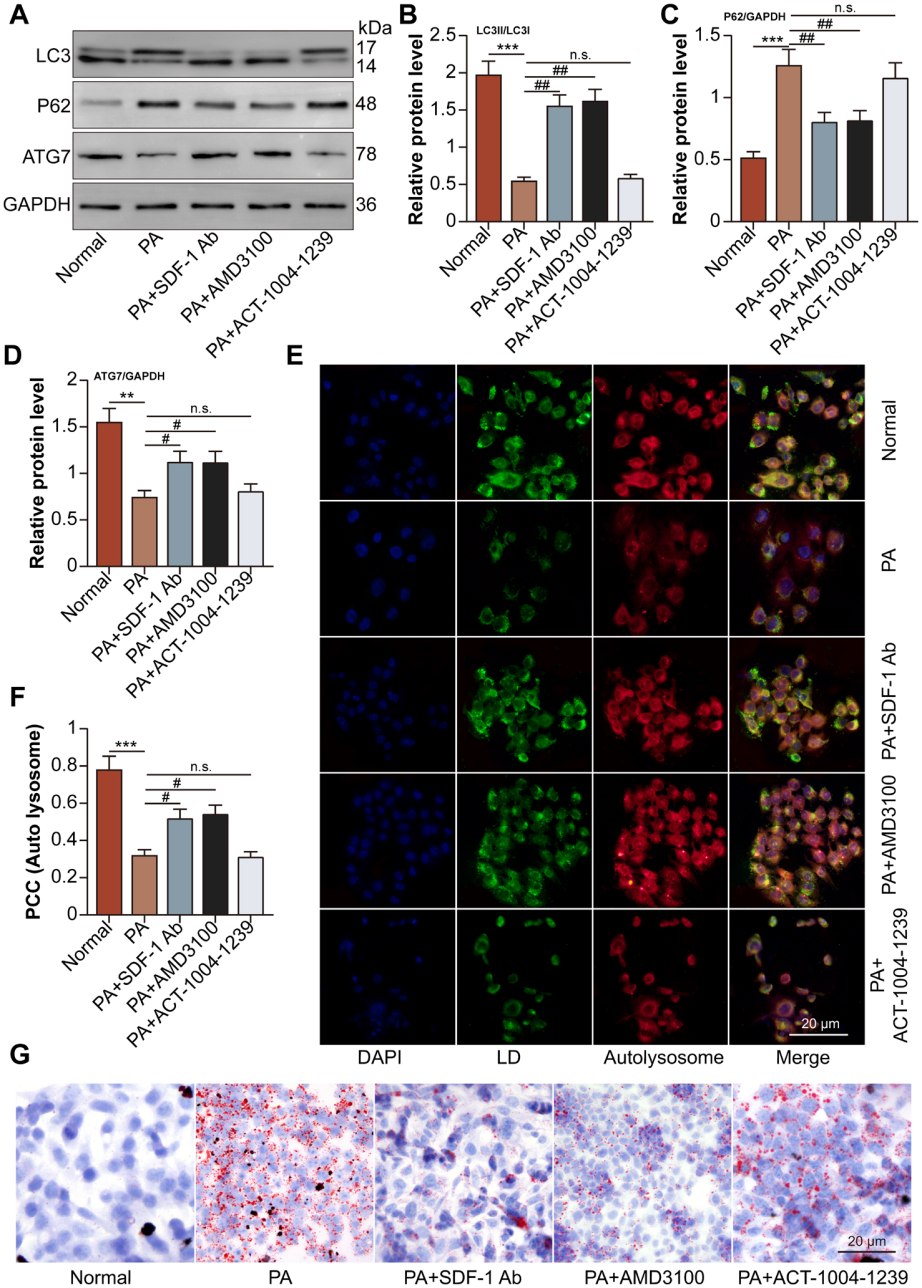
SDF‐1 inhibits lipophagy in PA‐treated hepatocytes via CXCR4, rather than CXCR7. The hepatocytes were divided into normal, PA, PA + SDF‐1 neutralising antibody (1 μg for 24 h), PA + AMD3100 (10 μM for 24 h), and PA + ACT‐1004‐1239 (6 nM for 24 h) groups. (A) LC3, p62 and ATG7 protein levels in hepatocytes were detected by Western blot. (B–D) LC3, p62 and ATG7 relative protein levels were analysed. (E) The co‐localization of the autolysosome (red) and the LD (green) in hepatocytes, indicated the induction of lipophagy (yellow). (F) The co‐localization of autolysosome and LD was analysed. (G) The LD inside the hepatocytes was visualised by oil red O staining. **p < 0.01, ***p < 0.001 versus normal group. ^#^ *p* < 0.05, ^##^ *p* < 0.01 versus PA group.

In normal groups, the hepatocytes displayed a high ratio of LC3II/LC3I, expression of ATG7, and co‐localization of autolysosome/LD, which were downregulated by PA treatment. Moreover, SDF‐1 neutralising antibody and CXCR4 antagonist AMD3100 up‐regulated the ratio of LC3II/LC3I (Figure 5A,B), the expression of ATG7 (Figure 5D), and of autolysosome/LD (Figure 5E,F) in PA‐treated hepatocytes, without the effect of CXCR7 antagonist ACT‐1004‐1239. The expression of p62 and lipid accumulation were induced by PA treatment in hepatocytes compared to those in normal groups. SDF‐1 neutralising antibody and AMD3100 down‐regulated the expression of p62 and lipid accumulation in PA‐treated hepatocytes, while ACT‐1004‐1239 unaffected the expression of p62 (Figure 5C) and lipid accumulation (Figure 5G).

### 3.6 SDF‐1/CXCR4 Inhibits Lipophagy in PA‐Treated Hepatocytes via Activating AKT/mTOR1 Pathway

**FIGURE 6:**
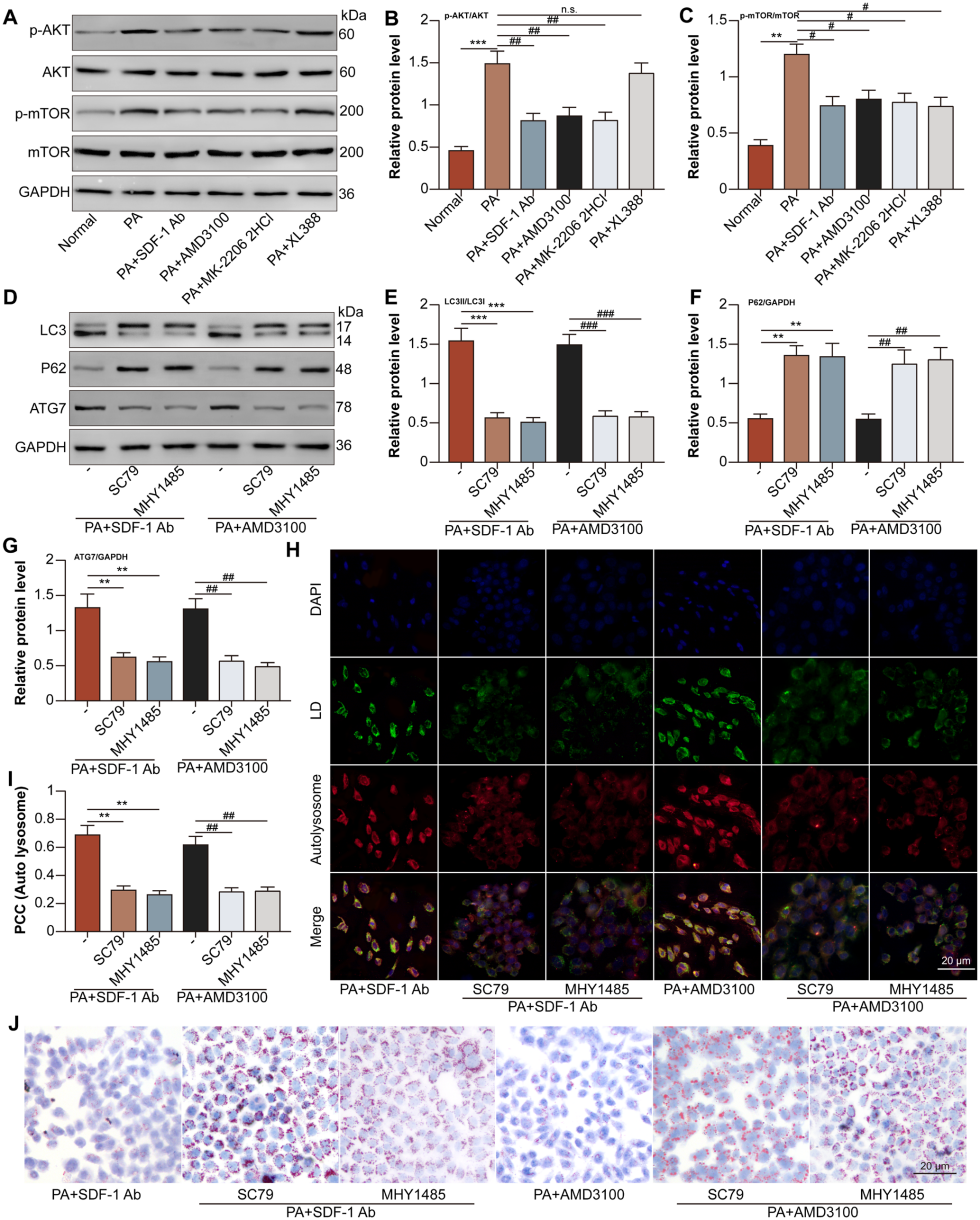
SDF‐1/CXCR4 inhibits lipophagy in PA‐treated hepatocytes via activating AKT/mTOR pathway. The hepatocytes were divided into normal, PA, PA + SDF‐1 neutralising antibody, PA + AMD3100, PA + MK‐2206 2HCl (10 μM for 24 h), and PA + XL388 (100 nM for 24 h) groups. (A) P‐AKT, AKT, p‐mTOR and mTOR protein levels in hepatocytes were detected by Western blot. (B, C) P‐AKT and p‐mTOR relative protein levels were analysed. In Figure 4B,C, **p < 0.01, ***p < 0.001 versus normal group. ^#^ *p* < 0.05, ^##^ *p* < 0.01 versus PA group. The hepatocytes were divided into PA + SDF‐1 neutralising antibody, PA + SDF‐1 neutralising antibody + SC79 (4 μg/mL for 24 h), PA + SDF‐1 neutralising antibody + MHY1485 (5 μM for 24 h), PA + AMD3100, PA + AMD3100 + SC79 and PA + AMD3100 + MHY1485 groups. (D) LC3, p62 and ATG7 protein levels in hepatocytes were detected by Western blot. (E–G) LC3, p62 and ATG7 relative protein levels were analysed. (H) The co‐localization of the autolysosome (red) and the LD (green) in hepatocytes, indicated the induction of lipophagy (yellow). (I) The co‐localization of autolysosome and LD was analysed. (J) The LD inside the hepatocytes was visualised by oil red O staining. In Figure 4E–G,I, **p < 0.01, ***p < 0.001 versus PA + SDF‐1 neutralising antibody group. ^##^ *p* < 0.01, ^###^ *p* < 0.001 versus PA + AMD3100 group.

The expression of p‐AKT and p‐mTOR in hepatocytes was induced by PA treatment, while SDF‐1 neutralising antibody, AMD3100 and AKT inhibitor MK‐2206 2HCl decreased the expression of p‐AKT in PA‐treated hepatocytes. However, mTOR inhibitor XL388 showed no effect on the expression of p‐AKT, indicating that mTOR was downstream of AKT. Meanwhile, SDF‐1 neutralising antibody, AMD3100 and XL388 decreased the expression of p‐mTOR in PA‐treated hepatocytes. Especially, MK‐2206 2HCl decreased the expression of p‐mTOR in PA‐treated hepatocytes, suggesting that AKT was upstream of mTOR (Figure 6A–C). High ratio of LC3II/LC3I (Figure 6D,E), expression of ATG7 (Figure 6G), and co‐localization of autolysosome/LD (Figure 6H,I) induced by SDF‐1 neutralising antibody and AMD3100 in PA‐treated hepatocytes were down‐regulated by AKT activator SC79 and mTOR activator MHY1485. Moreover, low expression of p62 (Figure 6F) and lipid accumulation (Figure 6J) inhibited by SDF‐1 neutralising antibody and AMD3100 in PA‐treated hepatocytes were up‐regulated by SC79 and MHY1485.

### 3.7 SDF1/CXCR4/AKT/mTOR Pathway‐Inhibited Lipophagy Promotes PA‐Induced Hepatocyte IR

**FIGURE 7:**
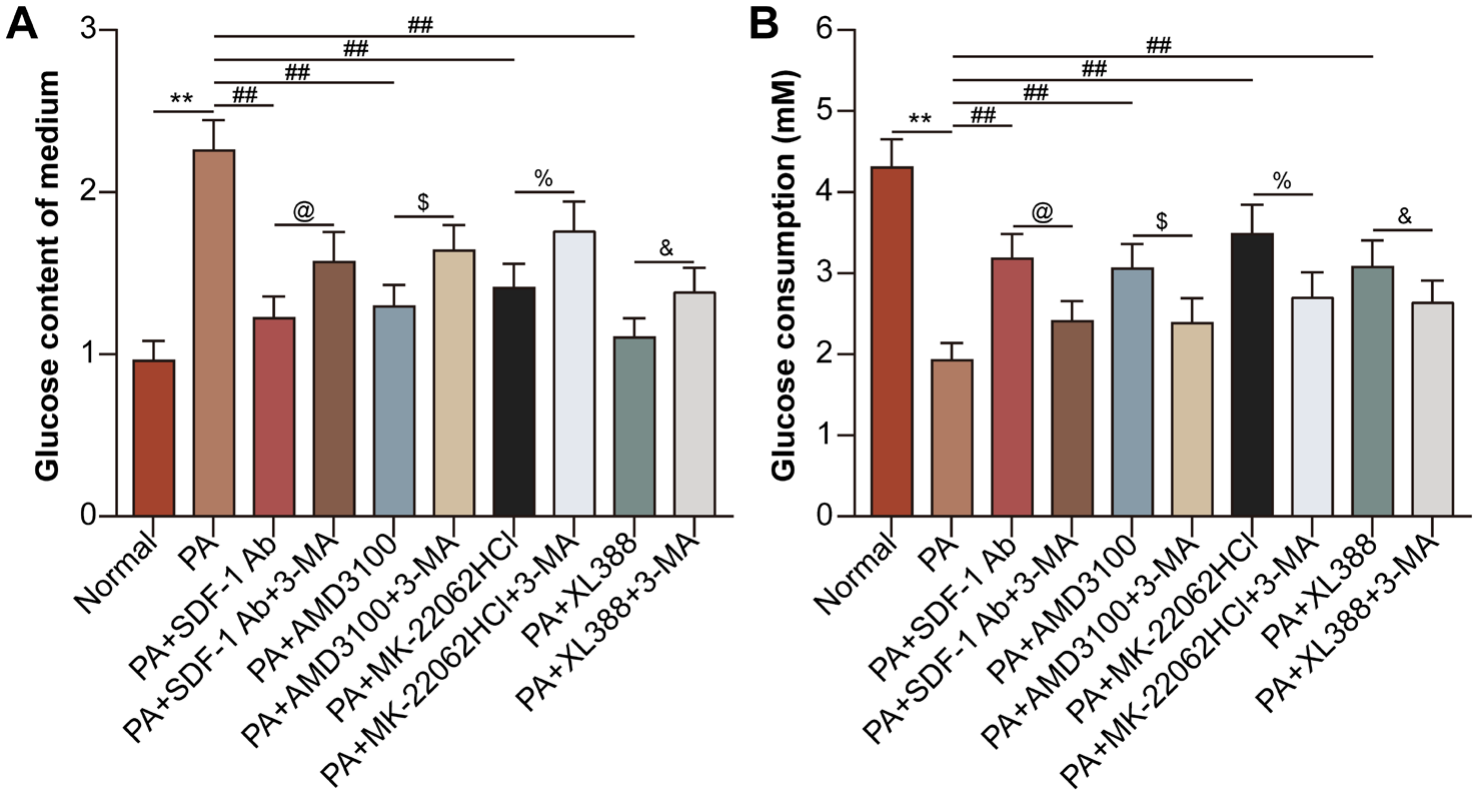
SDF1/CXCR4/AKT/mTOR pathway‐inhibited lipophagy promotes PA‐induced hepatocyte IR. The hepatocytes were divided into normal, PA, PA + SDF‐1 neutralising antibody, PA + SDF‐1 neutralising antibody +3‐MA (10 mM for 24 h), PA + AMD3100, PA + AMD3100 + 3‐MA, PA + MK‐2206 2HCl, PA + MK‐2206 2HCl + 3‐MA, PA + XL388 and PA + XL388 + 3‐MA groups. (A) The glucose content of the medium was measured. (B) Glucose consumption by hepatocytes was analysed. **p < 0.01 versus normal group. ^##^ *p* < 0.01 versus PA group. ^@^ *p* < 0.05 versus PA + SDF‐1 neutralising antibody group. ^$^ *p* < 0.05 versus PA + AMD3100 group. ^%^ *p* < 0.05 versus PA + MK‐2206 2HCl group. ^&^ *p* < 0.05 versus PA + XL388 group.

**FIGURE 8:**
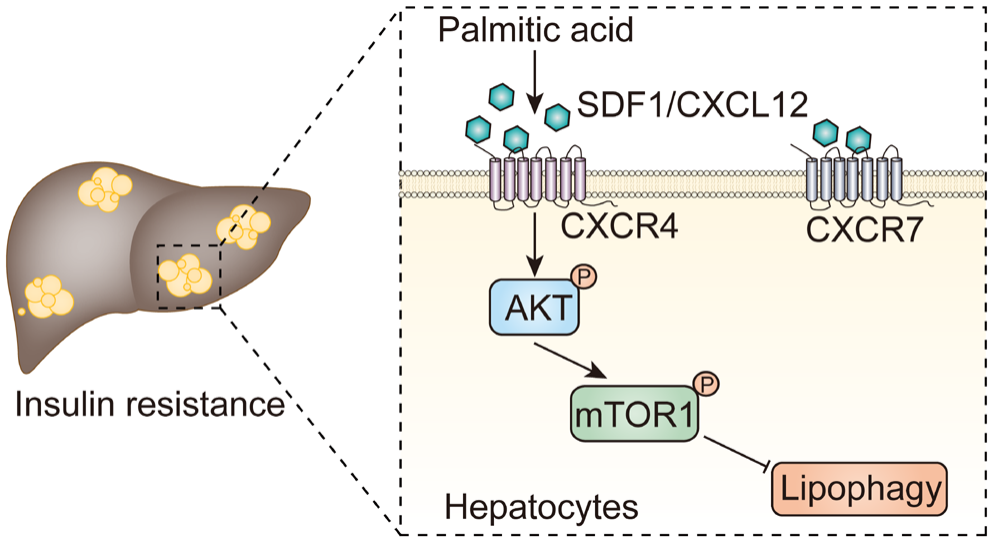
The role and mechanism of SDF‐1 in hepatic IR were displayed. Up‐regulated SDF1 binds to its receptor CXCR4 and CXCR7 on hepatocytes following PA treatment. SDF‐1/CXCR4 signalling, not SDF‐1/CXCR7 signalling, inhibits lipophagy in hepatocytes via activating the phosphorylation of AKT and mTOR1 to promote PA‐induced IR. The blockade of SDF‐1/CXCR4/AKT/mTOR signalling‐induced lipophagy alleviates IR in PA‐treated hepatocytes.

Glucose content in hepatocyte culture medium was low in normal group, while SDF‐1 neutralising antibody, AMD3100, MK‐2206 2HCl and XL388 decreased PA‐induced glucose content in hepatocyte culture medium. Autophagy inhibitor 3‐MA increased glucose content in PA‐treated hepatocyte culture medium suppressed by the inhibition of SDF‐1, CXCR4, AKT and mTOR (Figure 7A). The glucose consumption by hepatocytes showed the opposite tendency to that of glucose content in hepatocyte culture medium (Figure 7B). Finally, the role and mechanism of SDF‐1 in hepatic IR were shown in Figure 8.

## 4 Discussion

Firstly, we found that SDF‐1 expression in hepatocytes of mouse T2DM model increased. SDF‐1 produced by hepatocytes is up‐regulated by hypoxia [17], tumorigenesis [18] and toxic substance exposure such as carbon tetrachloride (CCl_4_) [19] and 3, 5‐diethoxycarbonyl‐1, 4‐dihydrocollidine [20]. We also found the neutralising of SDF‐1 alleviated IR and lipid accumulation in T2DM mouse liver via promoting lipophagy. Hepatic IR not only chronically up‐regulates gluconeogenesis, but also decreases insulin‐stimulated net glycogen synthesis and glucose uptake, in turn raising postprandial glucose production in a mouse T2DM model [21]. Knockdown of endogenous SDF‐1 in adipocytes obviously increases insulin sensitivity. Similarly, adipocyte‐specific ablation of SDF‐1 enhances insulin sensitivity in adipose tissues and in mouse whole body, indicating that SDF‐1 is an insulin‐desensitising factor in adipocytes [22]. Plasma SDF‐1 level increases in patients with T2DM [23], positively associated with diabetic insulitis [24], diabetic nephropathy [25], and adipose tissue inflammation [26].

Previous study demonstrates that PA up‐regulates the expression of SDF‐1 at the maternal‐foetal interface, potentially contributing to the prevention of recurrent implantation failure [27]. Consistent to this study, SDF‐1 expression in mouse hepatocytes following PA treatment increased in our study. More importantly, the release of SDF‐1 from PA‐treated hepatocytes also increased, which got us to think about whether SDF‐1 exerted its roles in an autocrine manner. Therefore, we detected the two receptors of SDF‐1, including CXCR4 and CXCR7 on hepatocytes following PA treatment. CXCR4 and CXCR7 expression on PA‐treated hepatocytes increased, suggesting that secreted SDF‐1 might bind to these two receptors on hepatocytes, which was authenticated by the interaction of SDF1/CXCR4 and SDF‐1/CXCR7. These data indicated that SDF‐1 played its role in an autocrine loop on PA‐treated hepatocytes. Oesophageal cancer stem cells (CSCs) secrete SDF‐1 and express CXCR4, which promotes the formation of an autocrine signalling loop, serving as a system to maintain the invasive, metastatic, and stem‐like properties of these CSCs [28]. Moreover, autocrine SDF‐1/CXCR4 signalling induces proliferation and survival, and invasion, promoting in vivo lung metastasis of CSCs for mouse advanced skin squamous cell carcinomas (SCCs) [29].

SDF‐1 shows double regulation role on autophagy. On one hand, cancer‐associated fibroblasts‐derived SDF‐1 inhibits autophagy in bladder cancer cells [30], while SDF‐1 from exosomes of mesenchymal stem cells (MSCs) inhibits ischemic myocardial cell autophagy [31]. On the other hand, SDF‐1 aggravates the progression of osteoarthritis by up‐regulating chondrocyte autophagy [32]. Additionally, SDF‐1 promotes the autophagy of Schwann cells [33]. These results indicate that SDF‐1 regulates autophagy dependent on distinct tissular, cellular and microenvironmental conditions. In 2009, it was revealed that autophagy can selectively degrade lipids in hepatocytes, which was termed as lipophagy [34]. The decreased hepatocyte lipophagy contributes to T2DM with lipid dysregulation through LD accumulation [35]. Herein, SDF‐1 inhibited lipophagy in PA‐treated hepatocytes, which is a novelty of our study. In previous studies, multiple phytochemicals have played their roles in lowering lipid via promoting lipophagy, such as kaempferol (flavonoid)^1^, and berberine (alkaloid)^2^. Thus, we speculated these phytochemicals target SDF‐1 in hepatocytes during T2DM.

SDF‐1 inhibits autophagy of human secretory phase endometrial stromal cells under oestrogen treatment conditions via CXCR4 [36]. CXCR4 at the ovine fetal‐maternal interface inhibits autophagy in the endometrium via CXCR4 [37]. CXCR7, also known as atypical chemokine receptor 3 (ACKR3), is expressed on multiple cell types, including hepatocytes [38]. CXCR7 acts as a scavenger to internalise and deliver SDF‐1 for lysosomal degradation [39]. This scavenging capability of CXCR7 is indispensable for sustaining the appropriate balance of SDF‐1 levels in the extracellular environment. Until now, the relationship between CXCR7 and autophagy is unclear. Our study discovered that SDF‐1 inhibited lipophagy in PA‐treated hepatocytes via CXCR4, rather than CXCR7.

SDF‐1 uses CXCR4 to activate AKT/mTOR pathway in mature dendritic cells [40]. Additionally, CXCR4 activation leads to increased phosphorylation of AKT/mTOR under hypoxic conditions in diffuse large B‐cell lymphoma (DLBCL) cell lines [41]. The activation of AKT/mTOR pathway promotes lipophagy in hepatocytes [42]. We found that SDF‐1/CXCR4 inhibited lipophagy in PA‐treated hepatocytes via activating AKT/mTOR pathway. However, whether other pathways, such as protein kinase AMP‐activated catalytic subunit alpha 2 (AMPK)^3,4^/nuclear factor kappa B (NF‐κB)^5^, participate in the downregulation of lipophagy by SDF‐1 needs further investigation.

Finally, the blockade of SDF‐1 alleviated PA‐induced hepatocyte IR. NOX‐A12 is a novel RNA aptamer that binds SDF‐1 in two key positions, blocking binding of the chemokine to its receptors and dislodging bound SDF‐1 from cell surfaces [79]. NOX‐A12 displays a synergistic action with programmed cell death 1 (PD‐1) blockade by enhancing T‐cell infiltration in heterotypic tumour‐stroma spheroids [43]. Thus, an exploratory phase 1B study with a small cohort of 11 colorectal and 9 pancreatic cancer patients has been performed, where a combination of NOX‐A12 with the PD‐1 inhibitor pembrolizumab induces T helper type 1 (Th1) immune responses and prolonged disease stabilisation in 25% of patients [44]. CXCR4 specific inhibitor AMD3100 [45] also showed the inhibitory role on PA‐induced hepatocyte IR. On 15 December 2008, the US Food and Drug Administration (FDA) approved AMD3100 use in combination with granulocyte‐colony stimulating factor (G‐CSF) to mobilise bone marrow haematopoietic stem cells (HSCs) to the peripheral blood for collection and subsequent autologous transplantation in patients with Non‐Hodgkin's lymphoma (NHL) or multiple myeloma (MM) [46]. Based on clinical applications, AMD3100 could be a novel drug to combat hepatic IR in T2DM.

In summary, the blockade of SDF‐1 ameliorated hepatic IR via promoting hepatocyte lipophagy in vivo. In vitro, up‐regulated SDF1 bound to its receptor CXCR4 and CXCR7 on PA‐treated hepatocytes. SDF‐1/CXCR4 signalling, not SDF‐1/CXCR7 signalling, inhibited lipophagy in hepatocytes via activating AKT/mTOR1 pathway to promote PA‐induced IR. The blockade of SDF‐1/CXCR4/AKT/mTOR signalling‐induced lipophagy mitigated IR in PA‐treated hepatocytes. The data supplied a potential molecular target SDF‐1 for the treatment of hepatic IR during T2DM. However, the activation and role of in vivo downstream CXCR4/AKT/mTOR pathway for SDF‐1 using SDF‐1 neutralising antibody (1 mg/kg/day) with CXCR4 activator CTCE‐0214 (25 mg/kg/d), AKT activator SC79 (10 mg/kg/d), or mTOR activator MHY1485 (10 mg/kg/d), intrahepatic injection; each reagent is given using intrahepatic injection, once a day from week 15 to week 16 of HFHSD feeding need further investigation. Moreover, in‐depth studies are warranted to test different doses and pharmacokinetics of SDF‐1 neutralising antibody, as well as the possible side effects of SDF‐1 inhibition.

## Author Contributions

**Chunfeng Lu:** validation (equal), writing – original draft (equal). **Yuting Zhang:** writing – original draft (equal). **Cuilian Sun:** validation (equal). **Yuhang Na:** investigation (equal). **Haotian Sun:** investigation (equal). **Jianhua Ma:** writing – review and editing (equal). **Xueqin Wang:** writing – review and editing (equal). **Xiaomin Cang:** project administration (equal), writing – review and editing (equal).

## Conflicts of Interest

The authors declare no conflicts of interest.

## Data Availability

The data that support the findings of this study are available from the corresponding author upon reasonable request.
